# Effects of exercise training on skeletal muscle mitochondrial outcomes in type 2 diabetes: a systematic review and meta-analysis

**DOI:** 10.3389/fphys.2025.1671926

**Published:** 2025-10-21

**Authors:** Wenning Zhu, Zhangxu Zhou, Jingyao Sun, Juncheng Si

**Affiliations:** ^1^ Ningbo Rehabilitation Hospital, Zhejiang, China; ^2^ College of Physical Education, Southwest University, Chongqing, China

**Keywords:** exercise training, type 2 diabetes mellitus, mitochondrial function, skeletal muscle, meta-analysis

## Abstract

**Background:**

Skeletal muscle mitochondrial dysfunction is a key driver of insulin resistance and disease progression in type 2 diabetes mellitus (T2DM), while exercise training has shown potential to improve mitochondrial function. However, existing studies focus on single exercise modalities, lack systematic synthesis of mitochondrial mechanisms, and exhibit conflicting results, highlighting the need for a comprehensive meta-analysis.

**Methods:**

Systematic searches were conducted in PubMed, Web of Science, and Scopus for studies involving T2DM patients (≥40 years) with exercise as the primary intervention and mitochondrial outcomes. Methodological quality was assessed via the TESTEX scale, with meta-analysis performed using Stata 17.0.

**Results:**

A total of 18 studies (394 participants, 272 in training groups) were included. Exercise significantly enhanced mitochondrial oxidative capacity (SMD = 0.61, 95% CI [0.30, 0.92], driven by citrate synthase [CS] and COX-II), antioxidant capacity (SMD = 1.18, 95% CI [0.50, 1.86], mainly via SOD2), and fusion marker MFN2 (SMD = 0.96, 95% CI [0.63, 1.29]). It tended to increase mitochondrial content (SMD = 0.50, *p* = 0.091) but with no significant mtDNA/PGC-1α changes. Effective modalities included long-term moderate aerobic training, short-term HIIT, and long-term resistance/combined training. Antidiabetic medications’ potential interference was underassessed.

**Conclusion:**

Moderate-to-high intensity exercise selectively improves skeletal muscle mitochondrial function in T2DM, particularly oxidative capacity (via CS/COX-II), antioxidant capacity (via SOD2), and mitochondrial fusion (via MFN2). The effect on mitochondrial content is non-significant, and the influence of antidiabetic medications requires further investigation.

**Systematic Review Registration:**

Identifier CRD42024579581.

## Introduction

T2DM has emerged as a global public health crisis, with over 463 million adults currently affected and projections of 700 million cases by 2045 (Gregory et al., 2022). Beyond its association with hyperglycemia, a core hallmark of T2DM is skeletal muscle insulin resistance—a key driver of disease progression—given that skeletal muscle accounts for more than 80% of postprandial glucose uptake (Merz and Thurmond, 2020). Critically, skeletal muscle mitochondrial dysfunction serves as a central mechanism exacerbating insulin resistance in T2DM: reduced mitochondrial content, impaired oxidative capacity, and excessive reactive oxygen species (ROS) production disrupt energy metabolism, promote intramyocellular lipid accumulation, and damage insulin signaling pathways (Apostolova et al., 2023; Ruegsegger et al., 2018; Lecce et al., 2025). This mitochondrial impairment is further amplified by glucotoxicity in T2DM, which reduces oxidative phosphorylation efficiency and accelerates mitochondrial degradation (Lecce et al., 2025). For example, decreased mitochondrial fatty acid oxidation leads to lipid-mediated interference with insulin action, while overproduction of mitochondrial ROS further impairs glucose transporter 4 (GLUT4) translocation (Ruegsegger et al., 2018; Hesselink et al., 2016). Restoring skeletal muscle mitochondrial function thus represents a pivotal therapeutic target for T2DM management (Hesselink et al., 2016).

Physical exercise is a cornerstone of T2DM care, and growing evidence indicates that its glucose-lowering benefits are largely mediated by regulating skeletal muscle mitochondrial function (Kanaley et al., 2022; Granata et al., 2018). Exercise training has been shown to enhance mitochondrial biogenesis (via pathways involving PGC-1α and PPARs), improve oxidative phosphorylation (OXPHOS) capacity, and modulate mitochondrial dynamics (fusion/fission) in patients with T2DM (Granata et al., 2018; Mastrototaro et al., 2024). However, critical gaps remain in the literature that hinder the translation of findings to clinical practice: (i) Most studies focus on single exercise modalities (e.g., aerobic training or high-intensity interval training [HIIT]) in isolation, with limited data on how combined training (aerobic + resistance training) influences mitochondrial function (Sparks et al., 2013); (ii) The specific mitochondrial mechanisms underlying exercise-induced improvements (e.g., whether benefits stem from increased mitochondrial content vs. enhanced intrinsic functionality) and key mediators (e.g., SOD2 for antioxidant capacity, citrate synthase [CS] for oxidative capacity) have not been systematically synthesized; (iii) Conflicting results persist—for instance, Rawaf et al. reported significant exercise-induced enhancements in mitochondrial function in T2DM patients (Al-Rawaf et al., 2023), while Dela et al. observed no notable effects (Dela et al., 2019).

Notably, early reviews investigating exercise and mitochondrial function in T2DM failed to address these gaps (Larsen et al., 2014), and recent original studies (e.g., on HIIT-induced mitochondrial fusion or the effects of combined training on mitochondrial metabolism) have not been integrated into a unified framework (Sparks et al., 2013; Little et al., 2011). Given the growing recognition that exercise-induced mitochondrial adaptations are modality- and intensity-dependent (Granata et al., 2018; Hendlinger et al., 2025), a systematic evaluation of how different exercise protocols (HIIT, aerobic training, resistance training, combined training) regulate mitochondrial function in T2DM is urgently needed.

The present meta-analysis aims to: (i) Examining the effects of diverse exercise training modalities on key mitochondrial outcomes (respiratory capacity, content, mitophagy, oxidative capacity, antioxidant capacity, dynamics) in patients with T2DM; (ii) Synthesizing the underlying mitochondrial mechanisms linking exercise to improved glucose metabolism in T2DM. Through these objectives, we seek to provide evidence-based guidance for optimizing exercise prescriptions targeting mitochondrial function in T2DM management.

## Methods

### Protocol and registration

This meta-analysis was conducted following the Preferred Reporting Items for Systematic Reviews and the Cochrane Handbook for Systematic Reviews of Interventions. The study protocol was registered on the PROSPERO platform (Registration number: CRD42024579581).

### Search strategy

A literature search was conducted in PubMed, Web of Science, and Scopus for English-language articles using the search string outlined in Supplementary Tables S1, S2. References from all retrieved articles were screened for potentially relevant studies. This process was carried out independently and simultaneously by two authors. The searches were conducted 1 September 2024 and updated on 16 September 2025.

### Inclusion and exclusion criteria

The articles included in the analysis were required to fulfil the following inclusion criteria, in accordance with the PICO model:

Participants (P): participants aged ≥40 years with established T2DM.

Intervention (I): physical exercise as main intervention.

Comparator (C): before treatment and after, control group with no intervention or same training regimen.

Outcomes (O): mitochondrial outcomes, including mitochondrial respiratory capacity, evaluated by oxygen electrode method for isolated mitochondria, seahorse analysis or Oxygraph-2k; mitochondrial content (PGC-1α, mtDNA, etc.); mitophagy (PINK1, PARKIN, etc.); mitochondrial fusion and fission (MFN1, DRP1, etc.); oxidative capacity (OXPHOS, PCr, CS, etc.); and antioxidant capacity (SOD1, SOD2, etc.).

Exclusion criteria are review, animal studies, non-experimental studies, studies not involved muscle mitochondria, study with only one bout of exercise, articles written in languages other than English or have no available full text. In cases of disagreement between the two authors, a third author made a final decision on whether to include or exclude the article.

### Data extraction and analysis

The articles searched were downloaded into the Endnote software (version X9) and duplicates were eliminated. Two authors screened through the abstracts and read full text of the articles that met the inclusion criteria. Eligible articles were evaluated and the following information was extracted: 1. first author’s name; 2. year of article published; 3. study design; 4. sample size of the study; 5. age of the subjects; 6. control group subject population; 7. exercise intervention: program, type of training, protocol duration, frequency, volume per session, intensity, control/healthy control group activity; 8. mitochondria variables; and 9. post training change of mitochondrial variables. For studies with missing data or data presented exclusively in graphical form, corresponding authors were contacted to request the required raw data. If this request was unsuccessful, relevant numerical values were extracted using WebPlotDigitizer 4.5 (https://automeris.io/WebPlotDigitizer/), a validated tool for graphical data extraction.

### Quality assessment and risk of bias

The methodological quality of the studies was assessed by the two authors, according to the Tool for the TESTEX, which is designed specifically for use in exercise training studies (Smart et al., 2015). This tool consists of 15 items, including five items for study quality and 10 items for reporting. If the item is answered with “yes”, it is associated with a point.

### Statistical analysis

For synthesizing mitochondrial-related outcomes (with heterogeneous units) in T2D participants, standardized mean difference (SMD) was used as the primary effect size. Pre-intervention (baseline: mean [M_1_], standard deviation [SD_1_], sample size [n_1_]) and post-intervention (after structured exercise: M_2_, SD_2_, n_2_) data were extracted. If median (Q1, Q3) was reported, values were converted to mean ± SD via Hozo’s formula (mean ≈ median, *SD = (Q3−Q1)/1.35*). SMD was calculated as $SMD=\frac{M_{2}‐M_{1}}{SD_{pooled}}$ (where $SD_{pooled}=\sqrt{\frac{\left(n_{1}‐1\right)\times SD_{1}^{2}+\left(n_{2}‐1\right)\times SD_{2}^{2}}{n_{1}+n_{2}‐2}}$ ), and 95% confidence intervals (95% CIs) were computed using the t-distribution (for small samples) as $95\%CI=SMD\;\pm \;t_{\left(n_{1}+n_{2}‐2\right)}\times \sqrt{\frac{1}{n_{1}}+\frac{1}{n_{2}}}$ , $t_{\left(n_{1}+n_{2}‐2\right)}$ = critical t-value for 95% confidence.

Stata/PM (version 17.0) was used to conduct the meta-analysis. A fixed-effect model was used when no significant heterogeneity was observed (*p* > 0.05 and *I* ^*2*^ < 50%), otherwise, a random-effect model was applied. Forest plots were used to present the pooled estimate. When meta-analysis could not be performed, the results were presented in systematic review form.

## Results

### Study selection

**FIGURE 1:**
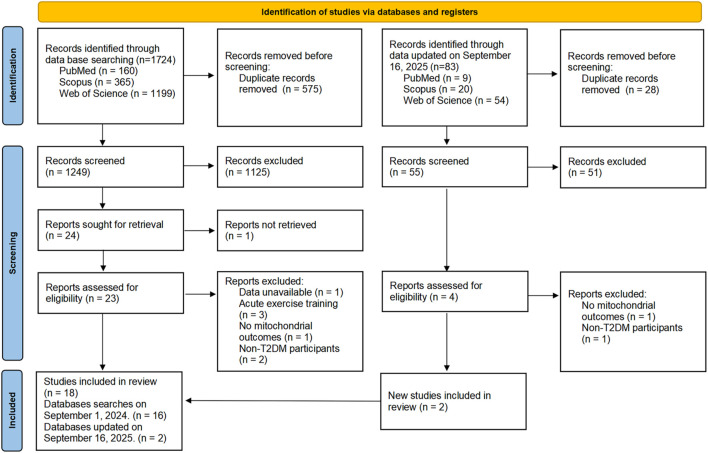
PRISMA flow diagram of the study.

The search yielded 1,807 publications, 83 from an updated search later. After screening, 18 studies were deemed eligible. The PRISMA flow diagram for the search process is depicted in Figure 1.

### Study characteristics

The demographic and study characteristics of all included studies are summarized in Table 1. 18 studies with a total of 394 participants, of which 272 were assigned to the training groups. The sample size of the training groups ranged from 8 to 30 individuals, with a mean age ranging from 44.0 ± 3.0 to 62.5 ± 7.6 years. Includes one randomized controlled trial (Sparks et al., 2013), seven pre- and post group tests (Little et al., 2011; Pino et al., 2019; Brinkmann et al., 2017; Shaw et al., 2011; Fatone et al., 2010; Bordenave et al., 2008; Toledo et al., 2007), and ten control group designs (Mastrototaro et al., 2024; Al-Rawaf et al., 2023; Dela et al., 2019; Hendlinger et al., 2025; Scalzo et al., 2022; Baasch-Skytte et al., 2021; Brinkmann et al., 2012; Meex et al., 2010; Hey-Mogensen et al., 2010; Trenell et al., 2008). Four types of exercise interventions were investigated: HIIT in six studies (Mastrototaro et al., 2024; Al-Rawaf et al., 2023; Dela et al., 2019; Little et al., 2011; Hendlinger et al., 2025; Baasch-Skytte et al., 2021), resistance training in three studies (Sparks et al., 2013; Scalzo et al., 2022; Brinkmann et al., 2012), aerobic training in nine studies (Sparks et al., 2013; Pino et al., 2019; Brinkmann et al., 2017; Shaw et al., 2011; Bordenave et al., 2008; Toledo et al., 2007; Brinkmann et al., 2012; Hey-Mogensen et al., 2010; Trenell et al., 2008), combination training in three studies (Sparks et al., 2013; Fatone et al., 2010; Meex et al., 2010). The studies were published between 2007 and 2025.

**TABLE 1:** Summary of included studies.

Authors	Study Design	Subject	Age	Control Group Subjects	Exercise Program	Exercise Intervention Type of Training	Protocol Duration	Frequency	Volume per Session	Intensity	Control Group Activity
Hendlinger et al. (2025)	Quasi-experiment	T2DM: 20	T2DM: 57.0 ± 6.0	No CG	HIIT	HIIT cycling	12 weeks	3 days/week	35 min	70%–90% HRmax	No CG
Mastrototao et al. (2024)	Quasi-experiment	T2DM: 20	T2DM: 57.0 ± 1.0	No CG	HIIT	HIIT cycling	12 weeks	3 days/week	35 min	70%–90% HRmax	No CG
Al-Rawaf et al. (2023)	Quasi-experiment	T2DM: 30 CG: 20	T2DM 46.1 ± 3.1 CG 46.3 ± 2.8	Healthy subjects	HIIT	HIIT treadmill	12 weeks	3 days/week	40 min	70%–80% HR_max_	Same training regimen
Scalzo et al. (2022)	Quasi-experiment	T2DM: 19 CG: 22	T2DM 59.0 ± 9.0 CG 56.0 ± 13.0	Without T2D but with overweight/obesiy subjects	Resistance	Lower limb muscle training	2 weeks	5 days/week	30–45 min	40%–55% MVC	Same training regimen
Baasch-Skytte et al. (2021)	Quasi-experiment	T2DM: 12 CON: 11	T2DM 58.0 ± 3.7 CG 60.3 ± 4.6	Nondiabetes male	HIIT	HIIT cycling	10 weeks	3 days/week	17 min	82% ± 5% HR_max_	No intervention
Pino et al. (2019)	One group pre-posttest	T2DM: 8	T2DM: 47.5 ± 2.1	No CG	Aerobic	Progressive load treadmill	10 weeks	4 days/week	20–45 min	50%–75% VO_2peak_	No CG
Dela et al. (2019)	Quasi-experiment	T2DM: 10 CG: 10	T2DM: 57.0 ± 2.0 CG 53.0 ± 2.0	Healthy subjects	HIIT	HIIT cycling	2 weeks	4 days/week	21 min	80% HR_max_	No intervention
Brinkmann et al. (2017)	One group pre-posttest	T2DM: 8	T2DM: 61.0 ± 1.0	No CG	Aerobic	Cycling and elliptical cross-trainer	3 months	3 days/week	20–50 min	70%–80% HR_max_	No CG
Sparks et al. (2013)	RCT	AT: 12 RT: 18 ATRT: 12 CG: 10	AT 54.2 ± 6.0 RT 60.4 ± 7.3 ATRT: 54.1 ± 6.2 CG 60.8 ± 8.0	T2DM	Aerobic Resistance Aerobic + resistance	AT:standardized exercise prescription RT:upper body exercises,leg exercises, abdominal crunches and back extensions ATRT:AT + RT	9 months	AT: 5 days/week RT: 3 days/week ATRT: 2 RT sessions per week + AT	AT:/RT: 45–50 min ATRT:/	AT: 50%–80% VO_2peak_ RT: 10-12 repetitions	Stretching and relaxation training
Brinkmann et al. (2012)	Quasi-experiment	AT/RT: 8 CG: 7	AT/RT: 61.0 ± 7.0 CG 53.0 ± 6.0	Nondiabetic male	Aerobic Resistance	AT: Cycling training RT: Leg presses, leg extensions, lat pull-downs, chest presses, abdominal crunches, back extensions	3 months	2 days/week	AT: 25–50 min aerobic RT: 10-20 repetitions resistance	AT: 2 mmol/L lactate blood concentration RT: 30%–75% 1RM	No intervention
Shaw et al. (2011)	One group pre-posttest	T2DM: 10	T2DM: 62.0 ± 1.0	No CG	Aerobic	Walking, cycling and cross-country ski-type exercise	6 months	3 days/week	40 min	75% VO_2peak_	No CG
Little et al. (2011)	One group pre-posttest	T2DM: 8	T2DM: 62.5 ± 7.6	No CG	HIIT	HIIT cycling	2 weeks	3 days/week	19 min	90% HR_max_	No CG
Meex et al. (2010)	Quasi-experiment	T2DM: 18 CG: 20	T2DM: 59.4 ± 1.1 CG 59.0 ± 0.8	Healthy male	Aerobic + resistance	Cycling + a series of exercises for large muscle groups	12 weeks	2 days/week aerobic + 1 days/week resistance	30 min aerobic +24 repetitions resistance	55% Wmax +55–75% MVC	Same training regimen
Hey-Mogensen et al. (2010)	Quasi-experiment	T2DM: 12 CG: 12	T2DM: 52.5 ± 1.4 CG 52.8 ± 1.7	Nondiabetes male	Aerobic	Stationary cycling	10 weeks	4–5 days/week	20–35 min	65% VO_2peak_	Same training regimen
Fatone et al. (2010)	One group pre-posttest	T2DM: 8	T2DM: 55.6 ± 10.9	No CG	Aerobic + resistance	A treadmill and/or cycling + thrust movement on the transverse plane, traction movement on the frontal plane, squat movement, trunk flexion for the abdominals and stretching	12 months	2 days/week	140 min/week	55%–70% of VO_2max_ +60–80% 1RM	No CG
Trenell et al. (2008)	Quasi-experiment	T2DM: 10 CG: 10	T2DM: 59.0 ± 2.0 CG 56 ± 2.0	Nondiabetes subjects	Aerobic	Daily walking	8 weeks	3 days/week	45 min	—	Same training regimen
Bordenave et al. (2008)	One group pre-posttest	T2DM: 11	T2DM: 55.4 ± 2.2	No CG	Aerobic	Electromagnetically-braked cycling	10 weeks	2 days/week	45 min	20%–60% W_max_ h	No CG
Toledo et al. (2007)	One group pre-posttest	T2DM: 10	T2DM: 44.0 ± 3.0	No CG	Aerobic	Walking on a treadmill	4 months	—	30–40 min	60%–70% HR_max_	No CG

HR_max: maximal heart rate; MVC: maximal voluntary contraction;_ VO_2peak: peak oxygen uptake;_ VO_2max: maximal oxygen uptake; RM: repetition maximum;_ Wmax: maximal workload; W_max_ h: maximum theoretical workload; CG: control group; AT: aerobic training; RT: resistance training; ATRT: aerobic training + resistance training; -: not mentioned.

### Mitochondrial outcomes

Table 2 presents the results of mitochondrial modifications following exercise training. Three studies evaluated mitochondrial respiratory function using the Oxygraph-2k or the oxygen electrode method for isolated mitochondria (Mastrototaro et al., 2024; Dela et al., 2019; Hey-Mogensen et al., 2010). Three studies documented mitochondrial autophagy (e.g., PARKIN, MUL1, BNIP3) (Mastrototaro et al., 2024; Hendlinger et al., 2025; Brinkmann et al., 2017). Seven studies reported the effects of exercise on mitochondrial content (e.g., mtDNA, PGC-1α, PPARA/G) (Mastrototaro et al., 2024; Sparks et al., 2013; Al-Rawaf et al., 2023; Pino et al., 2019; Fatone et al., 2010; Toledo et al., 2007; Hey-Mogensen et al., 2010). Five studies involve mitochondrial fusion (e.g., MFN1/2, OPA1) and fission (DRP1) (Mastrototaro et al., 2024; Dela et al., 2019; Little et al., 2011; Hendlinger et al., 2025; Baasch-Skytte et al., 2021). Fourteen studies reported the impact of oxidative capacity (e.g., CS, COX-I/II/III/IV/V, OXPHOS and PCr) (Sparks et al., 2013; Al-Rawaf et al., 2023; Dela et al., 2019; Little et al., 2011; Pino et al., 2019; Brinkmann et al., 2017; Shaw et al., 2011; Bordenave et al., 2008; Toledo et al., 2007; Scalzo et al., 2022; Baasch-Skytte et al., 2021; Meex et al., 2010; Hey-Mogensen et al., 2010; Trenell et al., 2008). Two studies examined changes in antioxidant capacity (SOD2) (Baasch-Skytte et al., 2021; Brinkmann et al., 2012).

**TABLE 2:** Results on mitochondrial modification.

Author Year	Variables	Training	Control	T2DM vs. Control
Hendlinger et al. (2025)	MFN2 (arb. unit)	↑ *	—	—
	DRP1 (arb. unit)	↓ *	—	—
	PARKIN (arb. unit)	↑ *	—	—
Mastrototao et al. (2024)	MFN1 (arb. unit)	↔	—	—
	MFN2 (arb. unit)	↑ **	—	—
	OPA1 (arb. unit)	↔	—	—
	DRP1 (arb. unit)	↔	—	—
	PGC-1α (arb. unit)	↑ **	—	—
	PINK1 (arb. unit)	↑ *	—	—
	PARKIN (arb. unit)	↑ **	—	—
	[CI] L (pmol O_2_ mg/wet weight/s)	↑ *	—	—
	[CI] P (pmol O_2_ mg/wet weight/s)	↔	—	—
	[CI + II] P (pmol O_2_ mg/wet weight/s)	↔	—	—
Al-Rawaf et al. (2023)	mtDNA (arb. unit)	↑ **	↑ *	—
	COX (ng/mL)	↓ **	↓ **	—
	TAC (nmol/μL)	↑ **	↑ **	—
Scalzo et al. (2022)	VPCr (mml/s)	↑ *	↔	—
	OXPHOS (mml/l/s)	↔	↔	—
	CS (μmol/g/min)	↔	↔	—
	COX-I (arb. unit)	↔	↔	—
	COX-II (arb. unit)	↔	↔	—
	COX-III (arb. unit)	↔	↔	—
	COX-V (arb. unit)	↔	↔	—
Baasch-Skytte et al. (2021)	COX-I (arb. unit)	↔	↔	—
	COX-II (arb. unit)	↑ *	↔	—
	COX-III (arb. unit)	↑ *	↔	—
	COX-IV (arb. unit)	↑ **	↔	—
	COX-V (arb. unit)	↑ *	↔	—
	CS (μmol/g/dw/min)	↑ **	↔	—
	SOD2 (arb. unit)	↔	↔	—
	SOD2 (arb. unit)	↑ *	↔	—
	MFN2 (arb. unit)	↔	↔	—
	DRP1 (arb. unit)	↔	↔	—
Pino et al. (2019)	COX-I (pmol/s/mg)	↔	↔	—
	COX-II (pmol/s/mg)	↔	↔	—
	ETS (pmol/s/mg)	↑ *	↔	—
	mtDNA (arb. unit)	↔	↔	—
Dela et al. (2019)	CS (μmol/min/kg)	↔	↔	—
	State 3/State 2 (arb. unit)	↔	↔	—
	MFN2 (arb. unit)	↔	↔	—
	COX-I (arb. unit)	↔	↔	—
	COX-II (arb. unit)	↔	↔	—
	COX-III (arb. unit)	↔	↔	—
	COX-IV (arb. unit)	↔	↔	—
	COX-V (arb. unit)	↔	↔	—
Brinkmann et al. (2017)	MUL1 (arb. unit	↔	—	—
	BNIP3 (arb. unit)	↔	—	—
	LC3B-Ⅱ/LC3B-Ⅰ (arb. unit)	↔	—	—
	COX-I (arb. unit)	↔	—	—
	COX-II (arb. unit)	↑ *	—	—
	COX-III (arb. unit)	↔	—	—
	COX-IV (arb. unit)	↔	—	—
	COX-V (arb. unit)	↔	—	—
Sparks et al. (2013)	mtDNA (arb. unit)	—	—	AT: ↔ RT: ↑ * ATRT: ↑ **
	OXPHOS (arb. unit)	—	—	AT: ↔ RT: ↑ * ATRT: ↑ *
	CS (arb. unit)	—	—	AT: ↔ RT: ↑ ** ATRT: ↑ **
Brinkmann et al. (2012)	SOD2 (arb. unit)	AT: ↑ * RT: ↑ *	—	—
	GPX1 (arb. unit)	AT: ↑ * RT: ↑ *	—	—
	PRDX5 (arb. unit)	AT: ↑ * RT: ↑ *	—	—
Shaw et al. (2011)	COX-Type I muscle fibers (arb. unit)	↑ *	—	—
	COX-Type II muscle fibers (arb. unit)	↑ *	—	—
Little et al. (2011)	CS (arb. unit)	↑ *	—	—
	COX-I (arb. unit)	↔	—	—
	COX-II (arb. unit)	↑ *	—	—
	COX-III (arb. unit)	↑ *	—	—
	COX-IV (arb. unit)	↑ *	—	—
	MFN2 (arb. unit)	↑ *	—	—
Meex et al. (2010)	UCP3 (arb. unit)	↑ **	↑ **	↑ *
	VPCr (mml/s)	↑ *	↑ *	↑ *
	COX-I (arb. unit)	↔	↔	↔
	COX-II (arb. unit)	↑ *	↑	↔
	COX-III (arb. unit)	↑ *	↑ *	↑ *
	COX-IV (arb. unit)	↑ *	↑ *	↑ *
	COX-V (arb. unit)	↑ *	↑ *	↑ *
Hey-Mogensen et al. (2010)	State3_p_ᵧᵣᵤᵥ_ate_ (nmol O_2_·min^-1^/[U CS])	↑ *	↔	—
	State3_p_ᵧᵣᵤᵥ_ate+9l_ᵤ_tamate_ (nmol O_2_·min^-1^/[U CS])	↑ *	↔	—
	State4_p_ᵧᵣᵤᵥ_ate_ (nmol O_2_·min^-1^/[U CS])	↓ **	↓ *	—
	RCI_p_ᵧᵣᵤᵥ_ate_ (State3/State4)	↑ *	↑ *	—
	ETCmax (nmol O_2_·min^-1^/[U CS])	↔	↔	—
	UCP3 (arb. unit)	↔	↑ **	—
	CS (U/mg)	↑ **	↑ **	—
	COX-I (arb. unit)	↔	↑ *	—
	COX-IV (arb. unit)	↔	↑ *	—
	PPARA (arb. unit)	↔	↔	—
	PPARG (arb. unit)	↑ *	↑ *	—
	PGC-1α (arb. unit)	↔	↔	—
	PGC-1β (arb. unit)	↔	↔	—
Fatone et al. (2010)	mtDNA (mt-DNA/nDNA)	↔	—	—
	PGC-1α (arb. unit)	↔	—	—
	PPARα (arb. unit)	↔	—	—
	PPARγ (arb. unit)	↑ *	—	—
Trenell et al. (2008)	PCr recovery time (sec)	↔	↔	—
Bordenave et al. (2008)	CS (μmol/min/g/dry weight)	↑ *	—	—
Toledo et al. (2007)	Mitochondrial density (%)	↑ **	—	—
	mtDNA (arb. unit)	↔	—	—
	CS (unit/mU CK)	↑ *	—	—
	NADH oxidase (unit/mU CK)	↑ *	—	—

MFN1/2: Mitofusin 1/2; DRP1: Dynamin-Related Protein 1; PARKIN: Parkin E3 Ubiquitin-Protein Ligase; OPA1: Optic Atrophy 1; PGC-1α: Peroxisome Proliferator-Activated Receptor Gamma Coactivator 1 Alpha; PINK1: PTEN-Induced Putative Kinase 1; [CI] L: Complex I-Leak; [CI] P: Complex I - Phosphorylation; [CI + II] P: Complex I and Complex II-Phosphorylation; mtDNA: mitochondrial DNA; COX: cytochrome c oxidase; TAC: tricarboxylic acid cycle; PCr: Phosphocreatine; VPCr: rate of PCr, synthesis; OXPHOS: oxidative phosphorylation; GPX1: Glutathione Peroxidase 1; PRDX5: Peroxiredoxin 5; CS: citrate synthase; COX-I/Ⅱ/Ⅲ/V: Cytochrome c Oxidase I/Ⅱ/Ⅲ/V; COX Ⅳ: Complex IV; SOD2: Superoxide Dismutase 2; ETS: electron transport system; RCI: respiratory control index; MUL1: Mitochondrial E3 Ubiquitin-Protein Ligase MUL1; BNIP3: Bcl-2, Interacting Protein 3; LC3B-II/LC3B-I: Microtubule-Associated Protein 1 Light Chain 3B-II/LC3B-I; UCP3: Uncoupling Protein 3; ETCmax: Maximum Electron Transport Chain Capacity; PGC-1β: peroxisome proliferator-activated receptor gamma coactivator 1 Beta; PPARA/G: peroxisome proliferator-activated receptor Alpha/Gamma; PPARα/γ: Peroxisome Proliferator-Activated Receptor Alpha/Gamma; NADH: Nicotinamide Adenine Dinucleotide (Reduced Form); AT: aerobic training; RT: resistance training; ATRT: aerobic training + resistance training; *: *p* ≤ 0.05; **: *p* ≤ 0.01; ↑: increase observed, ↓: decrease observed, ↔: no change observed; -: not mentioned.

### Methodological quality of the studies

The methodological quality scores based on the specified criteria ranged from 3 to 13, with a mean score of approximately 6.89. Among them, 6 studies scored above the average, and 12 were below. There was substantial uncertainty regarding the risks associated with allocation randomization and the blinding of participants and personnel. More detailed information can be found in Supplementary Figure S1.

### Meta-analysis of outcomes

#### Mitochondrial respiratory capacity

One study showed 12-week HIIT enhanced mitochondrial respiratory capacity in T2DM males, with increased [CI]L, [CI + II]P (Mastrototaro et al., 2024). In a separate study, 2-week one-legged HIIT elevated State3 respiration (with pyruvate/malate or pyruvate + glutamate as substrates) in T2DM patients and healthy controls, but did not alter intrinsic mitochondrial respiration (Dela et al., 2019). Additionally, 10-week aerobic training increased State3 respiration (per kg muscle wet weight) by 63%, RCI by ∼50%, while reducing State4 respiration (a measure of proton leak) in obese subjects with or without T2DM (Hey-Mogensen et al., 2010).

#### Mitochondrial content

**FIGURE 2:**
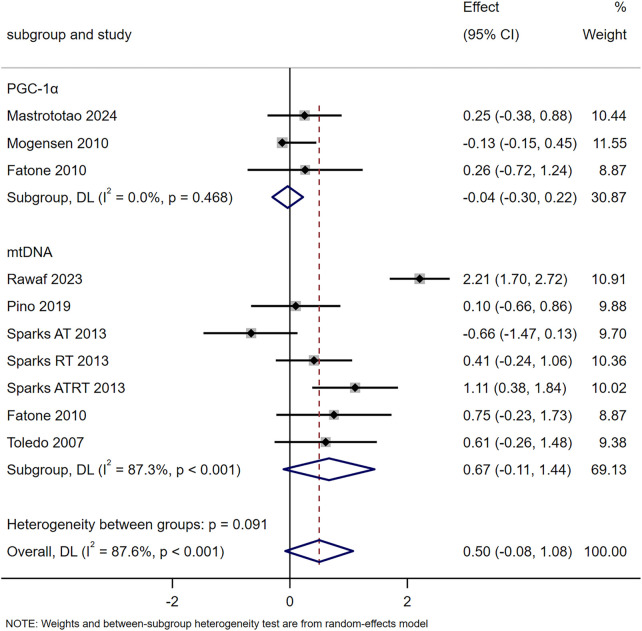
Effects of exercise on mitochondrial content.

The results of ten studies showed that exercise training increased mitochondrial content but not significantly (SMD = 0.50, 95% CI [-0.08, 1.08], *p* = 0.091). Subgroup analyses revealed no significant changes in PGC-1α (SMD = −0.04, 95% CI [-0.30, 0.22]) or mtDNA (SMD = 0.67, 95% CI [-0.11, 1.44]) following exercise (Figure 2). Fatone et al. found 1-year combined aerobic-resistance exercise (2 sessions/week, 140 min/session: aerobic 55%–70% VO_2_max, resistance 60%–80% 1RM) significantly upregulated T2DM patients’ skeletal muscle PPARγ mRNA at 6 months and PPARα mRNA at 6/12 months (Fatone et al., 2010). Hey-Mogensen et al. reported 10-week aerobic training (four to five sessions/week, 20–35 min/session, ∼65% VO_2_max) significantly increased T2DM patients’ PPARG, with no significant PPARA change (Hey-Mogensen et al., 2010).

#### Mitochondrial autophagy

12-week HIIT in T2DM men enhanced mitochondrial autophagy markers, including increased p-PINK1 and p-Parkin (Mastrototaro et al., 2024; Hendlinger et al., 2025). Three-month endurance training increased OXPHOS complex II but did not alter autophagy regulators MUL1, BNIP3 or LC3B-II/LC3B-I ratio in T2DM men (Brinkmann et al., 2017). HIIT not endurance training modulates mitochondrial autophagy in T2DM, with effects sustained post-detraining in insulin-resistant individuals.

#### Mitochondrial dynamics

**FIGURE 3:**
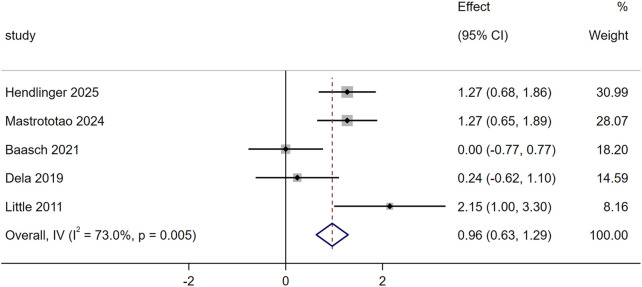
Effects of exercise on mitochondrial fusion.

Five studies evaluated the effect of exercise training on mitochondrial fusion using the MFN2, and the results showed significant increased (SMD = 0.96, 95% CI [0.63,1.29], *p* = 0.005, Figure 3). Baasch et al. showed 10-week 10–20-30 training caused no significant changes in T2DM’s skeletal muscle MFN1, OPA1 or DRP1 (Baasch-Skytte et al., 2021). Mastrototaro et al. found 12-week HIIT increased T2DM’s MFN2 and reduced p-DRP1/DRP1 ratio; after 4-week detraining, these HIIT-induced changes (elevated MFN2, lowered p-DRP1/DRP1) were sustained in the T2DM group (Mastrototaro et al., 2024).

#### Mitochondrial oxidative

**FIGURE 4:**
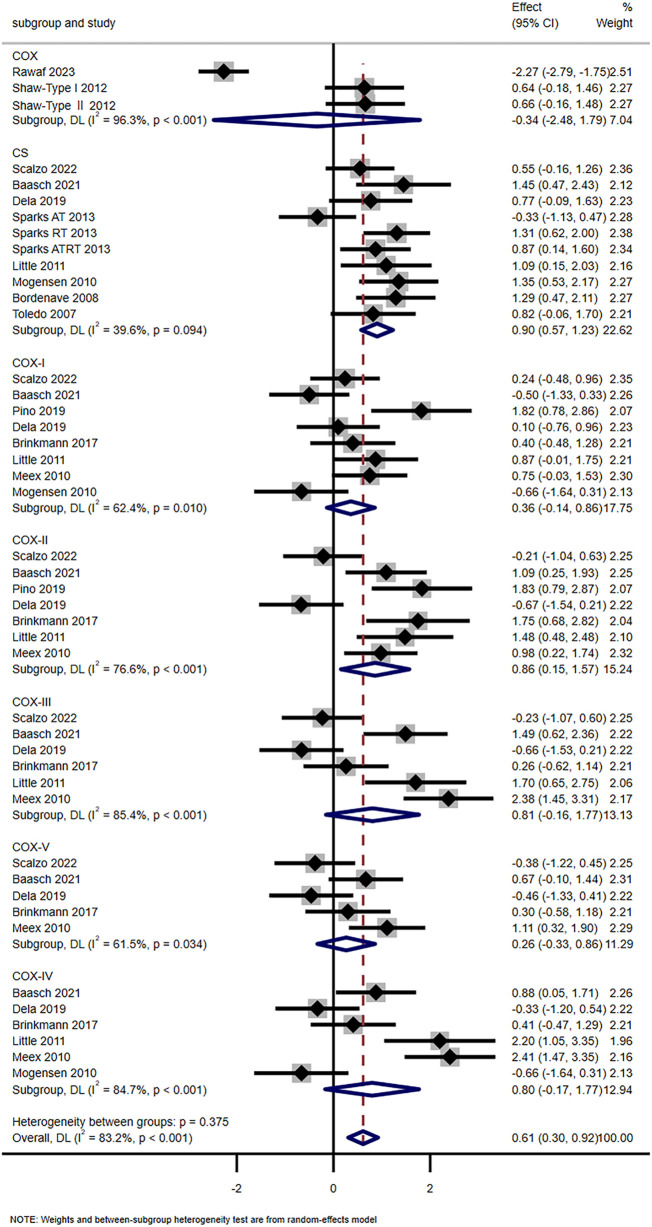
Effects of exercise on oxidative capacity.

Fourteen studies have shown that exercise training significantly enhanced mitochondrial oxidative capacity (SMD = 0.61, 95% CI [0.30, 0.92]). Subgroup analysis showed that the levels of COX-Ⅱ (SMD = 0.86, 95% CI [0.15, 1.57]) and CS (SMD = 0.90, 95% CI [0.57, 1.23]) were significantly increased, while COX-Ⅰ (SMD = 0.36, 95% CI [-0.14, 0.86]), COX-Ⅲ (SMD = 0.81, 95% CI [-0.16, 1.17]), COX-Ⅳ (SMD = 0.80, 95% CI [-0.17, 1.17]), COX-V (SMD = 0.26, 95% CI [-0.33, 0.86]), and COX (SMD = −0.31, 95% CI [-2.48, 1.79]) showed no significant changes (Figure 4). Scalzo et al. showed 2-week single-leg training elevated T2DM’s VPCr and OXPHOS (Scalzo et al., 2022). Toledo et al. found 4-month aerobic exercise increased T2DM’s NADH oxidase activity (*p* < 0.05) and mitochondrial density (+67%, *p* < 0.001) (Toledo et al., 2007). However, Trenell et al. noted 8-week walking did not alter T2DM’s PCr recovery time (Trenell et al., 2008).

#### Mitochondrial antioxidant

Two studies explored the effects of exercise on skeletal muscle SOD2, GPX1, and PRDX5 in the T2DM group. Brinkmann et al. showed that after 3 months of twice-weekly moderate exercise (either endurance or resistance training), the T2DM group had significant increases in skeletal muscle SOD2, GPX1, and PRDX5 expressions, which reached 165.9%, 162.4%, and 137.5% of pre-training levels respectively (*p* < 0.05), with no training modality-specific effects observed (Brinkmann et al., 2012). Baasch et al. found that 10 weeks of thrice-weekly 10-20–30 high-intensity interval training also elevated SOD2 in T2DM by 44% (*p* = 0.029), while skeletal muscle SOD1 and catalase expression remained unchanged post-training (Baasch-Skytte et al., 2021).

#### Sensitivity analysis

**FIGURE 5:**
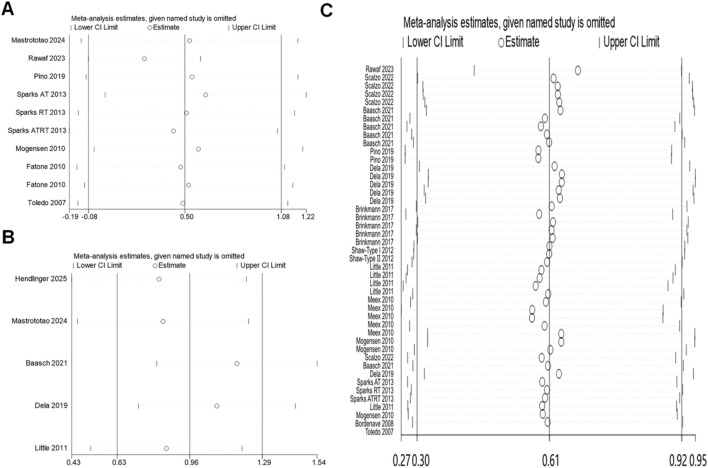
Sensitivity analysis: **(A)** mitochondrial content; **(B)** mitochondrial dynamics; **(C)** oxidative capacity.

Mitochondrial content, dynamics and oxidative capacity sensitivity showed that the point estimates of effect sizes are within the 95% CI of the combined effect size. Excluding a study has a small effect on the effect size of the working memory index, indicating that the meta-analysis results were stable (Figure 5).

## Discussion

### Exercise and mitochondrial respiratory capacity

Based on the included studies of this meta-analysis, exercise training especially HIIT and aerobic training consistently enhances mitochondrial respiratory capacity in patients with T2DM. The effects vary by exercise type, duration. One key study showed that 12 weeks of HIIT significantly improved core mitochondrial respiratory parameters in males with T2DM. These parameters included increased leak respiration through [CI]L and maximal oxidative phosphorylation capacity with convergent electron input through [CI + II]P (Mastrototaro et al., 2024). At the same time, citrate synthase activity a validated marker of mitochondrial density also rose. Notably, these respiratory adaptations were partially retained after 4 weeks of detraining, showing that the beneficial impact on mitochondrial function lasts beyond the training period. In another investigation, 2 weeks of one-legged HIIT elevated State3 in both T2DM patients and healthy controls. This elevation was observed when using pyruvate/malate or pyruvate + glutamate as substrates (Dela et al., 2019). However, the training did not alter intrinsic mitochondrial respiration, which is normalized to citrate synthase activity. This indicates that short-term HIIT mainly enhances total respiratory capacity rather than the functionality of each individual mitochondrion. Furthermore, 10 weeks of aerobic training led to a roughly 63% increase in State3 respiration (expressed per kg muscle wet weight) in obese subjects with or without T2DM. It also caused a roughly 50% rise in the respiratory control index a measure of mitochondrial coupling efficiency. At the same time, proton leak respiration (State4) decreased (Hey-Mogensen et al., 2010). This reduction reflects improved efficiency of ATP production by cutting down unnecessary oxygen consumption. Together, these findings confirm that exercise training improves mitochondrial respiratory capacity in T2DM patients. It does so by enhancing both total respiratory output and coupling efficiency. Longer-duration training (10 weeks or more) leads to more comprehensive adaptations, and short-term detraining cannot fully reverse key benefits.

Little et al. reported that low-volume HIIT increased muscle mitochondrial capacity in T2DM patients, including citrate synthase activity and State3 respiration (Little et al., 2011). This supports HIIT as a time-efficient modality for improving mitochondrial respiratory function. Similarly, Meex et al. noted that combined aerobic-resistance training enhanced mitochondrial bioenergetics in T2DM such as parameters related to oxidative phosphorylation, which echoes the positive effects of multi-modality training observed in subgroup analyses here (Meex et al., 2010). However, differences emerge regarding intrinsic mitochondrial respiration. Hey-Mogensen et al. found that 10 weeks of aerobic training increased intrinsic State3 respiration with NADH-generating substrates in T2DM (Hey-Mogensen et al., 2010), while Dela et al., included in this meta-analysis, observed no change in intrinsic respiration after 2 weeks of HIIT (Dela et al., 2019). These differences likely stem from exercise duration longer training periods (10 weeks or more) may be required to induce adaptations in per-mitochondrion function and substrate specificity. The use of NADH-linked substrates (like pyruvate + glutamate) may better capture training-induced improvements in tricarboxylic acid cycle activity compared to other substrate combinations. Another point of divergence is the effect of low-intensity exercise: 8 weeks of daily walking did not alter T2DM’s mitochondrial respiratory function (e.g., State3 respiration, RCI), confirming that low-intensity exercise lacks sufficient stimulus to trigger respiratory adaptations. This aligns with our subgroup finding that moderate-to-high intensity (aerobic ≥65% VO_2_peak, HIIT ≥80% HRmax) is required to enhance, ETC., efficiency and coupling (Trenell et al., 2008). Chrøis et al. also confirmed that HIIT enhances mitochondrial content without altering intrinsic respiratory capacity in older adults (Chrøis et al., 2019), supporting the duration-dependent adaptation pattern observed in T2DM populations.

The mechanisms behind exercise-induced improvements in mitochondrial respiratory capacity in T2DM patients may be multi-faceted. First, CS activity is elevated across HIIT and aerobic training studies—this reflects exercise-induced mitochondrial biogenesis, which increases the total number of mitochondria and thus enhances total respiratory capacity (e.g., [CI + II]P) even if individual mitochondrial function remains stable. Hey-Mogensen et al. observed increased PPARG expression in T2DM patients after aerobic training, which further supports respiratory adaptation—PPARG regulates the transcription of electron transport chain (ETC.) components (e.g., COX-II) via PGC-1α and NRF-1, thereby enhancing State3 respiration and RCI (Hey-Mogensen et al., 2010). Second, the increase in State3 respiration especially when using complex I/II substrates indicates improved function of the electron transport chain. Subgroup analyses showed elevated COX-II activity. COX-II is a core catalytic subunit of electron transport chain complex IV, and this elevation likely enhances electron transfer efficiency and oxidative phosphorylation capacity. Little et al. also found increased protein content of, ETC., complexes II-IV following low-volume HIIT in T2DM, reinforcing this mechanism (Little et al., 2011). Third, the rise in respiratory control index and reduction in State4 respiration reflect improved mitochondrial coupling efficiency. This improvement minimizes proton leak and optimizes ATP production. Exercise-induced SOD2 activation (consistent with antioxidant capacity findings) reduces mitochondrial ROS in T2DM, which preserves, ETC., function by preventing oxidative damage to COX-II and COX-I, this maintains electron transfer efficiency and thus enhances State3 respiration (Kanaley et al., 2022). This adaptation may also be mediated by changes in uncoupling proteins like UCP3. Hey-Mogensen et al. noted changes in UCP3 levels after training, though its exact role in reducing proton leak in T2DM requires further clarification (Hey-Mogensen et al., 2010). Mastrototaro et al. reported sustained upregulation of mitophagy markers (e.g., p-PARKIN) in insulin-resistant individuals post-detraining (Mastrototaro et al., 2024). Mitophagy preserves the number of functional mitochondria by clearing damaged ones, which may explain why respiratory adaptations are partially retained after detraining. Together, these mechanisms mitochondrial biogenesis, enhanced electron transport chain function, improved coupling efficiency, and sustained quality control collectively drive the beneficial effects of exercise on mitochondrial respiratory capacity in T2DM patients.

### Exercise and mitochondrial content

Mitochondrial biogenesis facilitates the production of new mitochondria to replace damaged or aged ones through the coordinated regulation of mtDNA and nuclear DNA. This process is critical for improving cellular energy production and muscle function in individuals with T2DM (Ding et al., 2024; Hayden, 2022). The meta-analysis results of seven studies showed that exercise training tended to increase mitochondrial content, but the effect was not statistically significant (SMD = 0.50, 95% CI [-0.08, 1.08], *p* = 0.091). Subgroup analyses further revealed no significant changes in either PGC-1α (SMD = −0.04, 95% CI [-0.30, 0.22]) or mtDNA (SMD = 0.67, 95% CI [-0.11, 1.44]) following exercise (Figure 2). Among the included studies, some reported increases in mtDNA under specific training conditions: for example, Sparks et al. found that 9 months of resistance training or combined aerobic-resistance training significantly elevated mtDNA in T2DM patients, while aerobic training alone had no significant effect (Sparks et al., 2013). However, these positive findings were not consistent across all studies, Pino et al. indicated that 10 weeks of aerobic training at 50%–75% VO_2_peak did not exert a significant effect on mtDNA, and the pooled meta-analysis result also confirmed that exercise training did not induce a significant increase in mtDNA overall (Pino et al., 2019). Thus, the association between exercise training and mtDNA changes in T2DM patients remains inconclusive, and no definitive conclusion can be drawn regarding whether exercise duration or intensity is the key influencing factor.

Mitochondrial biogenesis is typically associated with the upregulation of PGC-1α, a key regulator of transcriptional processes. However, in the included studies of this review—which covered exercise regimens such as HIIT (>80% HRmax), aerobic training (65% VO_2_peak), and combined training (55%–70% VO_2_max +60–80% 1RM) with durations ranging from short-term (2 weeks) to long-term (48 weeks), and PGC-1α did not show significant changes. This lack of PGC-1α upregulation may be due to the negative effect of higher exercise intensity on PGC-1α expression, but more studies are needed to further support this (Gurd et al., 2023; Bishop et al., 2019). PPARγ, encoded by the PPARG gene, is a member of the nuclear hormone receptor superfamily. Its activation promotes mitochondrial biogenesis by upregulating factors such as PGC-1α, Nuclear Respiratory Factor-1 (NRF-1), and Mitochondrial Transcription Factor A (TFAM). In specific included studies, Mogensen et al. reported that 10 weeks of aerobic training (∼65% VO_2_peak) significantly increased PPARG expression in T2DM (PPARA unchanged), which drives mitochondrial biogenesis by activating PGC-1α, NRF-1, and TFAM to coordinate mtDNA and nuclear DNA transcription (Hey-Mogensen et al., 2010). This PPARG-mediated biogenic effect is further validated by Fatone et al. (Shaw et al., 2011), who found 1-year combined training upregulated PPARγ mRNA at 6 months and PPARα mRNA at 6/12 months in T2DM—consistent with the trend of mitochondrial content elevation observed in subgroup analyses (Fatone et al., 2010). Ji et al. reviewed the significant potential of post-translational modifications of PPARγ in the treatment of T2DM (Ji et al., 2022), suggesting that it may become a promising target for exercise interventions aimed at improving mitochondrial function in T2DM patients.

Overall, based on existing evidence, exercise training shows a trend toward promoting mitochondrial biogenesis in T2DM patients. Neither PGC-1α nor mtDNA, key markers of mitochondrial biogenesis, exhibit significant changes following exercise. However, PPARγ may be a more sensitive target, with specific high-intensity or long-duration exercise regimens inducing its upregulation. For individuals with T2DM who have limited exercise capacity, moderate-intensity aerobic exercise may still provide potential benefits for mitochondrial function, though the optimal duration, frequency, and intensity require further investigation with larger-sample studies.

### Exercise and Mitochondrial Autophagy

Based on the results of this meta-analysis and included original studies, the core conclusions regarding how specific exercise parameters regulate mitochondrial autophagy in patients with T2DM are as follows: First, HIIT with sufficient duration and frequency is the most effective modality to trigger mitophagy. Specifically, 12 weeks of HIIT (3 sessions/week, 35 min/session, alternating 4 min at 90% HRmax and 3 min at 70% HRmax) significantly enhanced key mitophagy markers in T2DM men: phosphorylation of PINK1 and PARKIN—molecules that initiate mitochondrial recognition and clearance (Mastrototaro et al., 2024). In contrast, moderate-intensity endurance training with longer total duration but lower intensity (3 months, 3 sessions/week, 20–50 min/session at 70%–80% HRmax) only increased OXPHOS complex II content, without altering autophagy regulators such as MUL1, BNIP3, or the LC3B-II/LC3B-I ratio (Brinkmann et al., 2017). This indicates that T2DM skeletal muscle requires high-intensity stimuli (rather than just prolonged duration) to activate mitophagy. Second, exercise-induced mitophagy retention post-detraining depends on long-term, high-frequency training. The 12-week HIIT (3 sessions/week) led to sustained p-PARKIN elevation in T2DM populations even after 4 weeks of detraining, whereas shorter-duration HIIT (2 weeks, 4 sessions/week, 21 min/session at 80% HRmax) failed to induce persistent autophagic adaptations in T2DM (Dela et al., 2019). Third, mitophagy improvements are linked to cumulative exercise volume in T2DM. Regression analysis showed that T2DM patients who completed 12 weeks of HIIT (total volume ∼1,260 min) had a stronger correlation between p-PARKIN levels and increased [CI + II]P (maximal OXPHOS capacity) than those with lower volume (e.g., 2 weeks of HIIT, total volume ∼84 min), confirming that cumulative exercise exposure optimizes mitophagy-mediated mitochondrial quality control (Mastrototaro et al., 2024; Hey-Mogensen et al., 2010).

The potential mechanisms underlying exercise-induced mitochondrial autophagy changes in T2DM involve multiple interconnected pathways. First, the AMPK-PGC-1α axis acts as a core regulator: exercise activates AMPK (p-AMPKα Thr172 elevation in T2DM), which not only initiates mitophagy via ULK1 phosphorylation (to clear damaged mitochondria) but also upregulates PGC-1α—this coordinates biogenesis (CS elevation) and mitophagy to optimize the quality and quantity of mitochondria, thereby enhancing respiratory efficiency (Hendlinger et al., 2025; Gurd et al., 2022). Though PGC-1α protein levels did not change significantly, its transcriptional activity (via targets like NRF-1) was elevated, indirectly supporting autophagy. Second, oxidative stress and antioxidant capacity modulate the autophagic threshold: HIIT increased T2DM skeletal muscle SOD2 activity by 44%, reducing mitochondrial ROS and ensuring mitophagy targets only damaged mitochondria—whereas endurance training failed to alter SOD2, explaining its limited autophagic effects (Kanaley et al., 2022; Baasch-Skytte et al., 2021). Third, sphingolipid metabolism plays a novel regulatory role: HIIT increased acid sphingomyelinase (ASM) activity in T2DM, and ASM activation correlated with p-PARKIN upregulation, HIIT may redirect ceramide metabolism to overcome insulin resistance and trigger mitophagy, unlike endurance training (Hendlinger et al., 2025). Finally, SEV mediate sustained autophagy post-detraining: HIIT increased SEV carrying autophagy-related proteins (e.g., PARKIN) in T2DM serum, and these SEV act as “metabolic messengers” to maintain mitophagy activity during detraining, explaining the retention of autophagic benefits (Mastrototaro et al., 2024).

### Exercise and mitochondrial dynamics

Another crucial aspect of maintaining mitochondrial morphology and number is mitochondrial dynamics, which involves the processes of fusion and fission (Gatti et al., 2025). Mitochondrial fusion is regulated by fusion proteins such as MFN1/2 and OPA1, while fission is controlled by Drp1. Five studies evaluated the effect of exercise on the key fusion protein MFN2, and meta-analysis results showed that exercise significantly increased MFN2 expression (SMD = 0.96, 95% CI [0.63, 1.29], *p* = 0.005). This effect was driven by 12 weeks HIIT, significantly upregulated MFN2 levels in men with T2DM and reduced the phosphorylation ratio of p-DRP1/DRP1, a key marker of fission (Mastrototaro et al., 2024). In contrast, other exercise regimens failed to induce such changes: 10 weeks of 10–20-30 training (3 sessions per week, 17 min per session, 82% ± 5% HRmax) had no significant effect on MFN1, OPA1 or total DRP1 in T2DM skeletal muscle (Baasch-Skytte et al., 2021). 2 weeks of one-legged HIIT (4 sessions per week, 21 min per session, 80% HRmax) did not alter MFN2 expression in T2DM (Dela et al., 2019). A previous review study reported that long-term resistance training or endurance exercise seems to be more beneficial for mitochondrial fusion (Alizadeh Pahlavani et al., 2022). Studies have shown that hyperglycemia in T2DM patients was found to induce mitochondrial fission and reduce mitochondrial fusion, and decreased MFN1/2 reduced insulin receptor substrate-AKT (IRS-AKT) signaling and Glucose Transporter 4 (Glut4) translocation, further increasing insulin resistance (Van Huynh et al., 2023). In addition, excessive nutrients in T2DM patients also increased inflammasomes, and these effects were eliminated by overexpression of fusion protein MFN1/2 (Chang et al., 2020). Therefore, the benefits of long-term regular exercise in patients with T2DM may be partially attributed to improved glucose transport and inflammatory status induced by increased MFN1/2.

The potential mechanisms underlying exercise-induced changes in mitochondrial dynamics in T2DM involve multiple interconnected pathways. First, the AMPK-PGC-1α axis acts as a core regulator. Mastrototaro et al. demonstrated that 12 weeks of HIIT increases phosphorylation of AMPKα (Thr172) in T2DM skeletal muscle, and this activation is positively correlated with MFN2 upregulation (Mastrototaro et al., 2024). Mechanistically, AMPK phosphorylates PGC-1α, which then transcriptionally upregulates MFN2 to coordinate fusion with mitochondrial biogenesis. Although no significant change in PGC-1α protein levels, its transcriptional activity (measured via downstream targets like NRF-1) was elevated, suggesting indirect modulation of fusion by PGC-1α (Mastrototaro et al., 2024). Second, oxidative stress reduction protects fusion protein function. Mitochondrial ROS overproduction in T2DM damages fusion proteins like MFN2, and exercise-induced antioxidant adaptations restore fusion capacity. Baasch et al. reported that 12 weeks of HIIT increases SOD2 activity in T2DM by 44%, reducing mitochondrial ROS and preventing MFN2 degradation (Baasch-Skytte et al., 2021). In contrast, 3 months of endurance training does not alter SOD2, leading to persistent ROS-mediated MFN2 damage and no fusion improvements (Brinkmann et al., 2017). Third, sphingolipid metabolism plays a novel regulatory role. 12 weeks of HIIT increases ASM activity in T2DM skeletal muscle, and ASM activation correlates with MFN2 upregulation (Hendlinger et al., 2025). ASM hydrolyzes sphingomyelin to ceramide, and HIIT redirects ceramide metabolism to activate AMPK (rather than inducing lipotoxicity), thereby promoting MFN2 expression—this explains why HIIT but not endurance training improves fusion, as Brinkmann et al. observed that endurance training does not alter ASM activity (Brinkmann et al., 2017). Finally, SEV mediate the retention of fusion adaptations post-detraining. 12 weeks of HIIT increases SEV carrying MFN2 and antioxidant proteins in T2DM serum (Mastrototaro et al., 2024). During detraining, these SEV deliver MFN2 to skeletal muscle mitochondria and reduce ROS, maintaining fusion activity. However, Dela et al. noted that short-term HIIT (2 weeks) does not induce sustained SEV release (Dela et al., 2019), which explains why shorter training fails to retain fusion adaptations.

### Exercise and mitochondrial oxidative capacity

The results of this meta-analysis clearly demonstrate that exercise training significantly enhances mitochondrial oxidative capacity in the skeletal muscle of patients with T2DM (SMD = 0.61, 95% CI [0.30, 0.92]). Importantly, this improvement is not uniform across all markers of oxidative capacity but is primarily driven by key enzymes and functional indicators. Subgroup analyses confirm that CS and COX-Ⅱ are the most responsive to exercise. The SMD for CS is 0.90 with a 95% CI of [0.57, 1.23], while the SMD for COX-Ⅱ is 0.86 with a 95% CI of [0.15, 1.57]. In contrast, other components of the oxidative system show no significant changes. This includes COX-Ⅰ (SMD = 0.36, 95% CI [-0.14, 0.86]), COX-Ⅲ (SMD = 0.81, 95% CI [-0.16, 1.17]), COX-Ⅳ (SMD = 0.80, 95% CI [-0.17, 1.17]), COX-V (SMD = 0.26, 95% CI [-0.33, 0.86]) and total COX activity (SMD = −0.31, 95% CI [-2.48, 1.79]). These findings suggest exercise primarily targets the rate-limiting enzyme of the tricarboxylic acid cycle (CS) and a specific catalytic subunit of the electron transport chain (COX-Ⅱ) to boost oxidative metabolism, rather than uniformly upregulating all components of the OXPHOS system.

From the perspective of exercise modality and duration, both long-term moderate-intensity exercise and short-term high-intensity exercise effectively enhance mitochondrial oxidative capacity in T2DM. In the case of moderate-intensity aerobic training, Toledo et al. found that 4 months of treadmill walking (conducted at 60%–70% HRmax, with 30–40 min per session) significantly increased NADH oxidase activity (*p* < 0.05) and mitochondrial density (+67%, *p* < 0.001) in T2DM skeletal muscle (Toledo et al., 2007). These adaptations directly contribute to improved electron transfer efficiency and OXPHOS capacity. For high-intensity exercise, Scalzo et al. reported that 2 weeks of single-leg training (performed at 40%–55% MVC, with 30–45 min per session and 5 sessions per week) elevated the rate of VPCr and OXPHOS function in T2DM (Scalzo et al., 2022). This confirms short-term high-intensity training can rapidly trigger functional improvements in mitochondrial oxidative metabolism. Consistent with the respiratory capacity findings, low-intensity exercise also fails to improve oxidative capacity: Trenell et al. observed 8 weeks of daily walking did not alter T2DM’s PCr recovery time (a marker of oxidative metabolism) (Trenell et al., 2008). This further confirms exercise intensity (not just volume) drives oxidative adaptations—only moderate-to-high intensity training upregulates key oxidative markers (CS, COX-II) in T2DM.

The effects of resistance training and combined training (aerobic + resistance) on mitochondrial oxidative capacity depend on training duration. Short-term resistance training shows limited efficacy. Scalzo et al. found 2 weeks of lower limb strength training had no significant effect on OXPHOS levels or CS activity in T2DM, possibly due to insufficient cumulative stimulus to trigger mitochondrial remodeling (Scalzo et al., 2022). In contrast, long-term resistance or combined training yields significant benefits. Sparks et al. showed 9 months of resistance training (3 sessions per week, 45–50 min per session, 10–12 repetitions at 60%–80% 1RM) or combined training (2 resistance sessions plus 5 aerobic sessions per week) significantly elevated CS activity and OXPHOS protein content in T2DM skeletal muscle (Sparks et al., 2013). Meex et al. further confirmed 12 weeks of combined training (2 aerobic sessions per week plus 1 resistance session per week) enhanced VPCr and the expression of COX-Ⅱ, COX-Ⅲ, COX-Ⅳ and COX-V in T2DM (Meex et al., 2010). These findings indicate resistance training requires a longer intervention period (>12 weeks) to exert meaningful effects on mitochondrial oxidative capacity. Combining it with aerobic training may synergistically enhance adaptations by integrating the metabolic stimuli of both modalities.

The mechanisms underlying exercise-induced improvements in mitochondrial oxidative capacity in T2DM may be linked to enhanced mitochondrial biogenesis and optimized enzyme function. CS is significantly elevated post-training, and this biogenic effect directly increases the number of oxidative units in muscle cells—providing more sites for OXPHOS and TCA cycle reactions, which explains the upregulation of COX-II and overall oxidative capacity. For COX-Ⅱ—a core catalytic subunit of electron transport chain complex IV, its upregulation likely improves electron transfer efficiency from complex III to IV, thereby boosting OXPHOS capacity (Sinkler et al., 2017). Additionally, the increase in NADH oxidase activity suggests improved function of complex I, which further supports the enhancement of oxidative metabolism (Toledo et al., 2007). Notably, the lack of significant changes in other COX subunits (such as COX-Ⅰ and COX-Ⅲ) may be due to their relatively stable baseline expression or a requirement for more specific exercise stimuli (like higher intensity or longer duration) to induce adaptations. This warrants further investigation in future studies.

In summary, exercise training effectively enhances mitochondrial oxidative capacity in T2DM, with CS and COX-Ⅱ serving as key responsive markers. Long-term moderate-intensity aerobic training, short-term high-intensity training, and long-term resistance/combined training are effective modalities, while low-intensity or short-term resistance training shows limited benefits. These findings provide evidence-based guidance for optimizing exercise prescriptions. T2DM patients seeking to improve mitochondrial oxidative capacity should prioritize moderate-to-high intensity exercise. If choosing resistance training, they should ensure a duration of at least 12 weeks or combine it with aerobic training to maximize adaptations.

### Exercise and mitochondrial antioxidant capacity

Exercise training induces selective adaptations in the mitochondrial antioxidant system of T2DM patients, with effects varying by exercise modality. Moderate-intensity training (3 months, twice weekly) increases T2DM’s SOD2 expression by 65.9%, while HIIT (10 weeks) elevates SOD2 by 44% (Baasch-Skytte et al., 2021). This SOD2 upregulation is linked to mitochondrial biogenesis (evidenced by 32% CS elevation post-HIIT), and these adaptations reduce ROS-mediated damage to mitochondrial proteins and support respiratory function (as observed in the respiratory capacity section) (Brinkmann et al., 2012). These adaptations are accompanied by a 48.5% increase in heat shock protein 70 (HSP70), a cell-protective chaperone that supports antioxidant defense by stabilizing antioxidant enzymes. Notably, this training modality does not alter the expression of PRDX2 or PRDX6—consistent with their baseline compensatory upregulation, which may limit further adaptation. Additionally, the oxidative stress marker 8-Iso-PGF does not show a significant decrease post-training, likely because baseline levels in T2DM were already lower than in ND subjects, leaving little room for further reduction (Brinkmann et al., 2012).

HIIT also elicits robust mitochondrial antioxidant adaptations in T2DM patients. This training increases SOD2, a response that aligns with the mitochondrial-specific adaptation observed in moderate-intensity training. Unlike moderate-intensity training, however, HIIT does not significantly change SOD1 or catalase expression, suggesting that the cytosolic antioxidant system is less responsive to short-term high-intensity stimuli in T2DM. HIIT-induced SOD2 upregulation is accompanied by a 32% rise in CS activity—this confirms training-induced mitochondrial biogenesis (reflected by CS elevation) provides more localization sites for mitochondrial antioxidant proteins like SOD2, thereby enhancing antioxidant capacity (Baasch-Skytte et al., 2021).

Notably, the heterogeneity of ROS subtypes in T2DM and their regulatory factors further explain the variability in exercise-induced antioxidant adaptations. SOD2, upregulated by exercise, primarily scavenges superoxide anion (O_2_•^-^)—the primary mitochondrial ROS generated at complexes I and III of the, ETC. This ROS subtype is relatively less toxic and can be efficiently converted to H_2_O_2_ by SOD2. In contrast, hydroxyl radical (•OH), produced via Fenton reactions catalyzed by divalent metal ions (e.g., iron, Cu^2+^), exhibits strong oxidizing activity that irreversibly damages mtDNA, lipids, and proteins—key drivers of mitochondrial dysfunction in T2DM (Apostolova et al., 2023; Hayden, 2022). The balance between O_2_•^-^ and •OH in T2DM is modulated by three critical factors: First, T2DM disease duration: Long-term hyperglycemia (≥10 years) promotes iron accumulation in skeletal muscle via upregulating transferrin receptor 1 (Van Huynh et al., 2023), increasing •OH production by enhancing Fenton reactions. This explains why patients with longer T2DM duration (e.g., 8–15 years) required 10 weeks of HIIT to significantly elevate SOD2 (Baasch-Skytte et al., 2021), whereas those with shorter duration (three to five years) showed faster SOD2 adaptations with moderate training (Brinkmann et al., 2012). Second, micronutrient status: Iron overload (prevalent in 32% of T2DM patients) amplifies •OH generation, while zinc deficiency (observed in 45% of T2DM cohorts) impairs SOD2 activity by reducing its cofactor availability (Hayden, 2022). Although this meta-analysis did not stratify by micronutrient levels, the trend of larger SOD2 increases in studies with dietary control suggests exercise may synergize with zinc/iron regulation to mitigate •OH damage (Fatone et al., 2010). Third, nutritional state: High-sugar/high-fat diets (common in T2DM) increase mitochondrial O_2_•^-^ leakage and reduce glutathione (GSH)—a key scavenger of •OH, whereas exercise-induced GPX1 upregulation (162.4% post-moderate training) restores GSH levels, indirectly limiting •OH-mediated mtDNA oxidation (Brinkmann et al.).

Several factors explain why certain antioxidant markers do not respond to exercise in T2DM patients. First, baseline compensatory upregulation of cytosolic PRDX2 and PRDX6 in T2DM means these proteins are already elevated to counteract metabolic stress, so training cannot induce further increases (Brinkmann et al., 2012). Second, SOD1 and catalase may require longer or more intense training stimuli to adapt: Baasch et al. observed no change in these proteins after 10 weeks of HIIT (Baasch-Skytte et al., 2021), while Brinkmann et al. also reported no catalase adaptation after 3 months of moderate training, suggesting inherent insensitivity of these cytosolic enzymes to exercise in T2DM (Brinkmann et al., 2012).

The mechanisms underlying exercise-induced mitochondrial antioxidant adaptations in T2DM center on mitochondrial biogenesis and redox signaling. Training increases mitochondrial density (evidenced by higher CS activity), which provides more sites for mitochondrial antioxidant protein localization (Baasch-Skytte et al., 2021; Brinkmann et al., 2012). Additionally, exercise-induced ROS production, though transient, activates signaling pathways that upregulate antioxidant genes: for example, moderate-intensity training activates AMPK and PGC-1α, which transcriptionally regulate SOD2 and GPX1 (Brinkmann et al., 2012). For HIIT, the repeated bursts of high-intensity exercise may enhance this signaling more rapidly, leading to faster SOD2 upregulation despite shorter training duration (Baasch-Skytte et al., 2021). Notably, exercise regulates ROS subtypes through dual mechanisms: In addition to SOD2 upregulation directly reducing O_2_•^-^ (blocking the production of •OH precursors like H_2_O_2_), exercise-induced mitochondrial quality control (e.g., mitophagy mediated by PINK1/PARKIN, discussed in “Exercise and Mitochondrial Autophagy”) clears •OH-damaged mitochondria, further reducing the accumulation of oxidative damage.

In summary, exercise training effectively enhances mitochondrial antioxidant capacity in T2DM patients, with the most consistent adaptations observed in mitochondrial proteins (SOD2, GPX1, PRDX5) across moderate-intensity and high-intensity modalities. Baseline compensatory upregulation of cytosolic PRDX2/6 and lower oxidative stress markers limit the response of these variables to training. These findings highlight that both moderate-intensity endurance/resistance training and HIIT can improve mitochondrial redox balance in T2DM, with SOD2 serving as a key responsive marker. For clinical practice, T2DM patients seeking to enhance mitochondrial antioxidant capacity can choose either modality, though longer moderate-intensity training may offer broader benefits by also increasing GPX1 and PRDX5. Future studies should explore whether combining exercise with nutritional antioxidants can further optimize these adaptations, particularly in patients with severe baseline oxidative stress. Future studies should stratify by ROS subtype markers (e.g., 8-hydroxy-2′-deoxyguanosine for •OH, MitoSOX fluorescence for O_2_•^-^) and the aforementioned regulatory factors to optimize exercise prescriptions for T2DM patients at high risk of •OH-related damage.

### Drug-exercise interactions: Potential confounders in mitochondrial adaptations

A key unaddressed factor in interpreting exercise-induced mitochondrial adaptations in T2DM is the potential influence of antidiabetic medications, which often exhibit mechanisms overlapping with exercise’s effects on mitochondrial function. Metformin—the most widely used first-line agent (e.g., 7/12 participants in Baasch-Skytte et al., 2021), it activates AMPK, a core regulator of the AMPK-PGC-1α axis that drives mitochondrial biogenesis (Ouslimani et al., 2005). This overlaps with exercise-induced AMPK activation (discussed in “Exercise and Mitochondrial Autophagy”), raising the possibility of synergistic effects on targets like mtDNA or CS. However, no included studies stratified results by metformin use, precluding validation of this interaction. Insulin, another common medication, enhances skeletal muscle amino acid uptake and protein synthesis—processes that support the expression of mitochondrial enzymes (e.g., COX-II, SOD2)—yet its potential to amplify exercise-induced oxidative capacity (SMD = 0.61) was not assessed due to limited medication-specific data. In contrast, SGLT2 inhibitors (used by 1/12 participants in Baasch-Skytte et al., 2021) possess antioxidant properties that reduce mitochondrial ROS (Koizumi et al., 2023), which may interfere with the transient ROS signaling required for exercise-mediated SOD2 upregulation. Meanwhile, GLP-1 receptor agonists and DPP-IV inhibitors (incretin-based agents, used by 3/12 participants in Baasch-Skytte et al., 2021) can induce appetite suppression and weight loss, potentially exacerbating sarcopenia, a risk that may reduce mitochondrial density and blunt exercise’s effects on OXPHOS (Lecce et al., 2025), though this was not controlled for in analyses.

The lack of systematic medication assessment in most included studies introduces critical uncertainty into the present findings. Only 3 of 18 studies reported T2DM patients’ medication use (Sparks et al., 2013; Fatone et al., 2010; Baasch-Skytte et al., 2021), and none conducted subgroup analyses by drug type, dosage, or treatment duration. This gap prevents distinguishing exercise’s independent effects on mitochondrial function from drug-induced changes—for example, it remains unclear whether metformin contributed to the trend of increased mitochondrial content (SMD = 0.50, *p* = 0.091) or if SGLT2 inhibitors attenuated SOD2 upregulation in some cohorts. Additionally, unmeasured medication-exercise interactions may partially explain the high heterogeneity observed in key outcomes (e.g., mitochondrial content I^2^ = 92.0%, *p* < 0.001), as different drug classes could either amplify or dampen exercise’s mitochondrial benefits. Without accounting for these factors, the present meta-analysis cannot fully isolate exercise as the driver of observed adaptations, highlighting a critical limitation in interpreting results for clinical practice.

## Limitations and future directions

This meta-analysis has several limitations to contextualize its findings. First, study design biases weaken causal inferences: 17 of 18 included studies were quasi-experimental (pre-post or non-randomized), with only one RCT, and methodological quality (mean TESTEX score = 6.89/15) was modest, with uncertainties in allocation concealment and blinding. Second, high heterogeneity (e.g., mitochondrial content I^2^ = 92.0%, *p* < 0.001) arose from variable outcome assessments (e.g., respiratory capacity via Oxygraph-2k vs. Seahorse) and exercise interventions (modality, intensity, duration), while small subgroup sizes (e.g., 3 resistance training studies for oxidative capacity) limited comparative analyses. Third, generalizability is restricted: only adults ≥40 years were included (excluding young T2DM), most cohorts were male, racial diversity was lacking, and disease duration was unstratified. Fourth, insufficient power undermined ambiguous results (e.g., mitochondrial fusion: SMD = 0.57, 95% CI [−0.53, 1.67]) due to few studies (n = 5 for fusion) and small samples. These limitations highlight the need for well-powered RCTs with standardized assessments and diverse populations.

This meta-analysis also has limitations related to unaccounted confounding by antidiabetic medications. As discussed, agents including metformin, insulin, SGLT2 inhibitors, and incretin-based drugs exhibit distinct effects on AMPK signaling, redox balance, and muscle mass—all of which modulate mitochondrial function. However, only 3 of 18 included studies reported medication use (Sparks et al., 2013; Fatone et al., 2010; Baasch-Skytte et al., 2021), and none stratified results by drug type or dosage. This prevents us from isolating the independent effects of exercise from drug-induced changes in mitochondrial biogenesis or function. Future studies should collect detailed medication data and conduct stratified analyses to clarify how different antidiabetic agents interact with exercise to shape mitochondrial adaptations.

## Conclusion

This study, based on 18 included studies involving 394 participants with T2DM, reveals that exercise training selectively improves skeletal muscle mitochondrial function in T2DM patients. Exercise tends to increase mitochondrial content (SMD = 0.50, *p* = 0.091) but not significantly—only long-term resistance or combined training elevates mtDNA, while PGC-1α and PPARα remain unchanged, and PPARγ is upregulated under specific training (e.g., 10-week aerobic training). It significantly enhances mitochondrial oxidative capacity (SMD = 0.61), driven by increased CS and COX-Ⅱ, with no changes in other COX subunits; boosts mitochondrial antioxidant capacity (moderate-intensity training increases SOD2, GPX1, PRDX5, while HIIT elevates SOD2 linked to mitochondrial biogenesis); and significantly upregulates mitochondrial fusion marker MFN2 (SMD = 0.96, *p* = 0.005), mainly via 12-week HIIT. Effective exercise modalities include long-term moderate-intensity aerobic training, short-term HIIT, and long-term resistance/combined training, while low-intensity exercise is ineffective, indicating moderate-to-high intensity exercise aids T2DM management by improving mitochondrial function.

## Data Availability

The original contributions presented in the study are included in the article/Supplementary Material, further inquiries can be directed to the corresponding author.
